# The Left Superior Longitudinal Fasciculus within the Primary Sensory Area of Inferior Parietal Lobe Plays a Role in Dysgraphia of Kana Omission within Sentences

**DOI:** 10.3233/BEN-2012-100147

**Published:** 2012-04-24

**Authors:** Nobusada Shinoura, Akira Midorikawa, Toshiyuki Onodera, Ryozi Yamada, Yusuke Tabei, Yasumitsu Onda, Chihiro Itoi, Seiko Saito, Kazuo Yagi

**Affiliations:** ^1^Department of NeurosurgeryKomagome Metropolitan HospitalBunkyo-kuTokyoJapan; ^2^Department of PsychologyChuo University of LiteratureHachioji CityTokyoJapan; ^3^Department of Radiologic TechnologyTokyo Metropolitan University of Health SciencesArakawa-kuTokyoJapan

**Keywords:** Brain tumor, DTI, dysgraphia, inferior parietal lobe, kana, SLF

## Abstract

Functional neurological changes after surgery combined with diffusion tensor imaging (DTI) tractography can directly provide evidence of anatomical localization of brain function. Using these techniques, a patient with dysgraphia before surgery was analyzed at our hospital in 2011. The patient showed omission of kana within sentences before surgery, which improved after surgery. The brain tumor was relatively small and was located within the primary sensory area (S1) of the inferior parietal lobe (IPL). DTI tractography before surgery revealed compression of the branch of the superior longitudinal fasciculus (SLF) by the brain tumor. These results suggest that the left SLF within the S1 of IPL plays a role in the development of dysgraphia of kana omission within sentences.

## 1. Introduction

Acquired dysgraphia occurs after focal brain damage of the left hemisphere, such as injury to the frontal, parietal or temporal lobes [1,2]. The Japanese writing system uses two different scripts, namely kana (phonetic writing) and kanji (morphograms), and Japanese sentences are usually written with a combination of kana and kanji. There are two pathways of language, i.e., the phonologic route and the morphologic route, which run from the temporal lobe through the parietal lobe to the frontal lobe via the ventral and dorsal systems, respectively [2,3]. Investigators have recently used diffusion tensor imaging (DTI) tractography to visualize the white matter pathways [4]. DTI detects diffusion of water along the axonal fibers to visualize the white matter pathways in the living human brain, which was reconstructed using a region of interest (ROI) approach including the superior longitudinal fasciculus (SLF) [5, 6].

Surgery for brain tumors can provide evidence of the anatomical localization of brain function by comparing neurological changes before and after surgery with the corresponding damaged cortex or white matter

ISSN 0953-4180/12/$27.50 © 2012 – IOS Press and the authors. All rights reserved

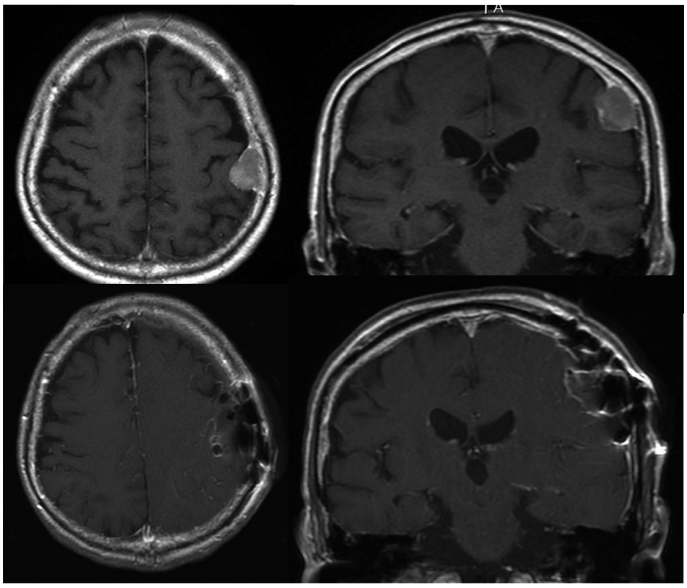

Fig. 1. Axial and coronal MRI with contrast enhancement before and after surgery. Axial (Panel 1) and coronal (Panel 2) MRI with contrast enhancement (Panel A, before surgery; Panel B, one week after surgery) shows brain tumor located in the left S1 of IPL, which was totally removed.

pathways represented on DTI tractography. The goal of the present study was to use this technique to analyze a patient with a relatively small brain tumor in order to determine the anatomic location critical for the development of dysgraphia.

## 2. Patient and methods

### *2.1. Patient*

In 2011, a patient underwent surgery for brain tumor at our hospital. The patient was a 79-year-old right-handed male who was a native speaker of the Japanese language. The patient completed 6 years of education (i.e., elementary school level) and did not have any prior history of neurologic or psychiatric disease.

The patient showed dysgraphia before surgery. The brain tumor was located within the primary sensory area (S1) of inferior parietal lobe (IPL) (Fig. 1).

DTI was performed pre-operatively to localize the SLF. Informed consent to perform DTI and surgery was obtained from the patient.

### *2.2. DTI and image analysis*

DTI and image analysis were performed as described previously [7]. Briefly, standard imaging gradients were used with a maximum strength of 23 mT/m and a slew rate of 50 mT/m/ms. The DTI acquisition sequence used single-shot spin-echo echo planar imaging with the following parameters: echo time, 127.6 ms; acquisition matrix, 128 $\times$ 128; and field of view, 24 cm $\times$ 24 cm [8]. Contiguous 5-mm-thick slices were acquired, covering the whole brain, with a b value of 1000 mm$^2$/s in 30 non-collinear directions. The reconstructed voxel size was 1.88 $\times$ 1.88 $\times$ 4.00 mm$^3$. DTI acquisition time for a total of 61 images was approximately 10 min. Diffusion tensor eigenvalues ($\lambda_1$, $\lambda_2$, $\lambda_3$) and eigenvectors ($\varepsilon_1$, $\varepsilon_2$, $\varepsilon_3$) were calculated from DTI data, and fractional anisotropy (FA) maps [5] were generated according to the Tensorlines (TL) algorithm. Tractography results were generated with the TL algorithm, as a combination of the Tensor Deflection (TEND) algorithm at low FA [9] and Streamlines tracking (STT) at high FA [10], using DTI Analyzer software (IDL version 5.6; Research Systems, Boulder, CO. A stopping criterion (threshold 0.1) was used for analysis.

The SLF was constructed using the ROI of the white matter of the coronal section, which showed a triangular shape just lateral to the corticospinal tract near the anterior horn of the lateral ventricle where the tracts ran from posterior to anterior. Of note, the SLF does not include the arcuate fasciculus. To assess the fronto-parietal SLF fibers only in DTI, we used one ROI, as described above.

Table 1
Summary of test results for language function before and after surgery

| Standardized test | Before surgery | After surgery |
|---|---|---|
| Fluency of spoken words (95) | 100 | 100 |
| Object naming (95) | 100 | 100 |
| Word repetition (98) | 95 | 95 |
| Verbal word fluency (55) | 30 | 45 |
| Sentence comprehension (88) | 88 | 88 |
| Kana reading (98) | 100 | 100 |
| Kanji reading (92) | 100 | 100 |
| Sentence (Kanji & Kana) writing for explanation of picture (65) | 3 | 53 |
| Sentence (Kanji & Kana) writing to dictation (50) | 45 | 100 |
| Kana writing (80) | 83 | 75 |
| Kanji writing (63) | 50 | 75 |

Language function of this patient was assessed as described in the Methods section. Values were calculated according to the instructions in WAB. The maximum score in each domain is 100. The cutoff score is shown in parentheses.

### *2.3. Tumor resection*

Awake surgery was performed as described previously [11]. Briefly, the patient was positioned in the supine lateral position with rigid head fixation (Mayfield headrest; Mizuho Medical, Tokyo, Japan) after administration of local anesthetic agents (1% xylocaine and 0.75% anapain) at pin sites and regional field block sites. Under intravenous anesthesia with propofol and remifentanil, the skin was infiltrated with the same local anesthetic agent and incised, and neuronavigated craniotomy and incision of the dura was performed. The patient was continuously observed by the neurosurgeon and a neurologist.

The tumor was removed in the usual fashion. Adequacy of language function was continuously assessed during tumor removal [11]. Tumor removal was assisted by neuronavigation. Following completion of tumor resection, intravenous anesthesia was administered using propofol. After closure of the dura, the bone flap was replaced, and the skin was closed in the usual manner.

## 3. Neuropsychological testing

The Japanese version of Western Aphasia Battery (WAB) was performed to measure language ability before and 1 week after surgery [12]. Fluency of spoken words, object naming, sentence comprehension, word repetition, verbal word fluency, kana or kanji reading, and kana, kanji or sentence (combination of kana and kanji) writing were assessed using the WAB. To assess general intellectual functioning before and after surgery, Raven's Coloured Progressive Matrices (RCPM) was performed [13].

## 4. Results

### *4.1. Language function before, during and after surgery in the patient*

Language function before or after surgery in the patient is summarized in Table 1. The patient showed no definite abnormalities of language function except writing and verbal word fluency before surgery. During awake surgery, he did not show any deterioration of language function. One week after surgery, he showed improvement in verbal word fluency from 30 to 45, sentence writing for explanation of a picture from 3 to 53, sentence writing to dictation from 45 to 100 and kanji writing from 50 to 75 (Table 1). Regarding writing before surgery, although the patient made well-formed graphemes of kana and kanji characters, the most serious deficit of writing was the omission of some kana characters in a sentence, namely, 80% of nouns and verbs. These results indicate that the omission of kana characters within sentences occurred before surgery, and drastically improved 1 week after surgery. In terms of general intellectual functioning, the RCPM score slightly improved after surgery (20/36) when compared with the preoperative score (17/36).

### *4.2. MRI before and after surgery, and tractography before surgery*

Before surgery, a relatively small brain tumor ($2 \times 2.5 \times 3$ cm) compressed the brain in the S1 of the IPL (Fig. 1A). After surgery, the compression of the brain was relieved due to the total removal of the brain tumor (Fig. 1B). Preoperative tractography revealed that the left SLF ran just around the tumor in the S1 of the IPL (Fig. 2). One branch of the SLF (white arrow in Fig. 2) was highly compressed by the tumor (white arrowhead in Fig. 2).

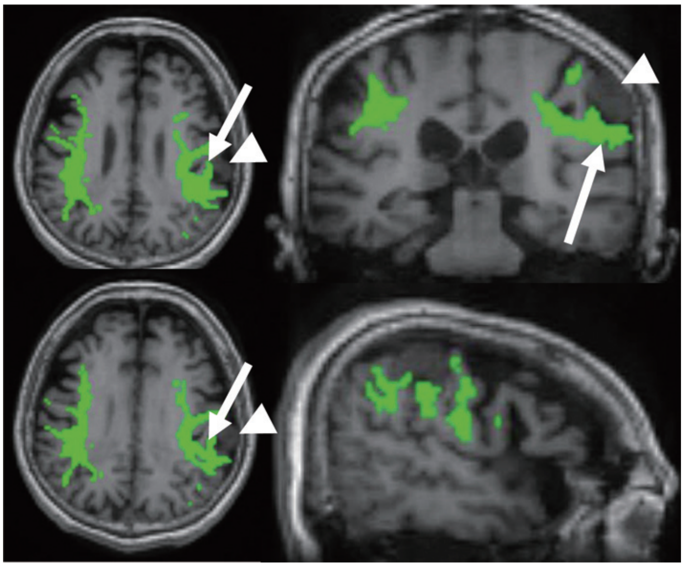

Fig. 2. DTI images of the SLF before surgery. The SLF (green) was constructed in axial (Panel 1) and coronal (Panel 2) MRI as described in the Methods section. The left SLF ran around the tumor (white arrowhead), and one branch of the left SLF (white arrow) was highly compressed by the tumor.

## 5. Discussion

The present study described the case of a patient with dysgraphia before surgery, which improved after surgical removal of the brain tumor. Since the brain tumor was relatively small (∼2 to 3 cm in diameter), it compressed a small region within the S1 of the IPL (Fig. 1). Before surgery, the most serious impairment of writing was the omission of kana characters within sentences, and the patient was able to make well-formed graphemes of kana and kanji characters. Notably, words written in kana characters are generally much easier to write than words written in kanji characters. The deficit in kana characters drastically improved 1 week after surgery. In fact, the patient's writing score for kana script only was better when compared with the kanji writing score prior to surgery. The kana writing score decreased slightly following surgery, although the score remained within normal limits before and after surgery. These results indicate that omission of kana characters occurred only when the patient attempted to write a combination of kana and kanji within sentences. Tractography before surgery revealed that the tumor directly compressed the part of the SLF passing subjacent to S1 of the IPL along with the cortical regions interconnected by the SLF, which is possibly third branch (Fig. 2) [14]. These results suggest that the compression of the left SLF in the S1 of the IPL plays a role in the omission of kana characters within sentences (i.e., a symptom of dysgraphia). Notably, after surgery, kanji writing improved, kana writing performance fell below cutoff score, mixed kana/kanji sentence writing to dictation became completely normal, and sentence writing for picture explanation, while much improved, still remained impaired, while the pre-surgery mixed kana/kanji writing score for pictures was 3 out of 100. These results suggests that kanji writing performance must have been impaired for sentence writing as well before surgery, although the score of kanji writing performance before surgery (score; 50) fell just below cutoff score (score; 63). This might suggest that the compression of the left SLF in the S1 of the IPL slightly affect the kanji writing performance.

Investigators have previously utilized lesion analysis in patients with cerebral infarction, bleeding, and brain tumors to demonstrate that various lesions are associated with dysgraphia, indicating that lesions for dysgraphia are distributed in a widespread fashion from the frontal to occipital lobe. Agraphia usually co-occurs with aphasia but occasionally presents as an isolated deficit. This observation led to a proposal for a distinct centre for writing (i.e. Exner's centre) in the posterior part of the second frontal gyrus [15].The left posterior inferior temporal cortex has been reported to store memory representations for the written forms of familiar words [16]. Since writing is executed by using the motor area of the frontal lobe, memory of written words may be conveyed from the temporal lobe to frontal lobe via nerve tracts, although there are no DTI studies to help characterize the pathological mechanisms of dysgraphia. The SLF is a probably candidate for such a tract, because it connects the temporal lobe and occipital lobe with the frontal lobe via the parietal lobe [6]. Indeed, lesions associated with dysgraphia seem to be related to dorsal systems, such as the dorsolateral frontal lobe, centrum semiovale or angular gyrus [1,17,18], and the main nerve tract in the dorsal system is the SLF [19]. Therefore, damage to the SLF in the IPL might disrupt transmission of word images from the posterior inferior temporal lobe to the parietal or frontal lobe, resulting in dysgraphia [20]. Notably, there are several types of dysgraphia, including apraxic dysgraphia. In this case, since the patient made well-formed graphemes of kana and kanji characters, this type of dysgraphia was not apraxic dysgraphia, which usually occurs secondary to damage of the superior parietal lobe (SPL) [21,22]. On the other hand, in this case, information of graphemes of kana and kanji was normally conveyed in the SPL, but the information of kana characters within sentences, which might be stored in the S1 of the IPL, might not be conveyed via the SLF secondary to compression by the brain tumor. Moreover, the patient was not impaired in other skills such as drawing. Since various short connections were recently reported about the sensory-motor integration, further investigation is required to determine the lesion and mechanism responsible for the various types of dysgraphia including this case [23].

The left SLF may mediate other functions in addition to writing. Indeed, the left SLF is mainly associated with language-related function, including reading, and speed of lexical decision or cognitive processing [19,24–27]. All these language-related functions may be associated with writing, since these functions can improve with writing. In addition to language-related function, the left SLF is associated with spatial working memory [28]. Since the left SLF mainly conveys higher-level language-related function, such as cognition, lexical decision or writing, damage to the left SLF may induce impairment of thinking and result in psychiatric disease. Indeed, various psychiatric diseases are associated with abnormalities of the left SLF. DTI studies revealed that abnormalities of the left SLF are associated with a working memory deficits and auditory hallucinations in the context of schizophrenia, major depression and autism spectrum disorder [29–34]. Therefore, when undertaking surgical resection of brain tumors, care should be taken to avoid damage to nerve fibers (such as the left SLF) during tumor resection. Several advanced neurosurgical techniques, including awake surgery and the ability to utilize tractography data along with neuronavigation, may facilitate the preservation of nerve tract integrity [35]. Finally, it is to be noted that although we found relatively clear results in this study, there are limitations in specific and case studies in general like this study.

In conclusion, these results suggest that the left SLF within the S1 of the IPL plays a role in the omission of kana characters within sentences.

## Acknowledgments

This work was supported by Foundation of Tokyo Metropolitan Hospitals.
